# CD36 Contributes to Malaria Parasite-Induced Pro-Inflammatory Cytokine Production and NK and T Cell Activation by Dendritic Cells

**DOI:** 10.1371/journal.pone.0077604

**Published:** 2013-10-28

**Authors:** Nagaraj M. Gowda, Xianzhu Wu, Sanjeev Kumar, Maria Febbraio, D. Channe Gowda

**Affiliations:** 1 Department of Biochemistry and Molecular Biology, Pennsylvania State University College of Medicine, Hershey, Pennsylvania, United States of America; 2 Molecular Cardiology, Lerner Research Institute, Cleveland Clinic, Cleveland, Ohio, United States of America; INRS - Institut Armand Frappier, Canada

## Abstract

The scavenger receptor CD36 plays important roles in malaria, including the sequestration of parasite-infected erythrocytes in microvascular capillaries, control of parasitemia through phagocytic clearance by macrophages, and immunity. Although the role of CD36 in the parasite sequestration and clearance has been extensively studied, how and to what extent CD36 contributes to malaria immunity remains poorly understood. In this study, to determine the role of CD36 in malaria immunity, we assessed the internalization of CD36-adherent and CD36-nonadherent *Plasmodium falciparum*-infected red blood cells (IRBCs) and production of pro-inflammatory cytokines by DCs, and the ability of DCs to activate NK, and T cells. Human DCs treated with anti-CD36 antibody and CD36 deficient murine DCs internalized lower levels of CD36-adherent IRBCs and produced significantly decreased levels of pro-inflammatory cytokines compared to untreated human DCs and wild type mouse DCs, respectively. Consistent with these results, wild type murine DCs internalized lower levels of CD36-nonadherent IRBCs and produced decreased levels of pro-inflammatory cytokines than wild type DCs treated with CD36-adherent IRBCs. Further, the cytokine production by NK and T cells activated by IRBC-internalized DCs was significantly dependent on CD36. Thus, our results demonstrate that CD36 contributes significantly to the uptake of IRBCs and pro-inflammatory cytokine responses by DCs, and the ability of DCs to activate NK and T cells to produce IFN-γ. Given that DCs respond to malaria parasites very early during infection and influence development of immunity, and that CD36 contributes substantially to the cytokine production by DCs, NK and T cells, our results suggest that CD36 plays an important role in immunity to malaria. Furthermore, since the contribution of CD36 is particularly evident at low doses of infected erythrocytes, the results imply that the effect of CD36 on malaria immunity is imprinted early during infection when parasite load is low.

## Introduction

Malaria, a devastating disease caused by the *Plasmodium* family of protozoan parasite species, is endemic in many parts of the tropical and subtropical regions of the world and inflicts enormous morbidity and mortality [1], [2]. Although several *Plasmodium* species cause malaria in humans, *P. falciparum* accounts for the majority of malarial deaths [3]. This is attributed to the ability of *P. falciparum* to sequester in the microvascular capillaries of organs such as brain, kidney and lungs, and in the blood space of placenta, contributing to cerebral, placental, and other organ-related severe malaria (reviewed in [4]–[6]). The sequestration is mediated by the binding of *P. falciparum* erythrocyte membrane protein 1 family of antigenically variant proteins, expressed by parasites on the surface of infected red blood cells (IRBCs), to different host receptors, including CD36, intercellular adhesion molecule-1 (ICAM-1), vascular adhesion molecule-1 (VCAM-1), and P-selectin on the endothelial cell surface, and chondroitin 4-sulfate (C4S) in the placenta [7]–[13] and (reviewed in [14]–[16]). In the case of mouse malaria, although the parasite ligand involved has not been identified, studies have shown that CD36 mediates the sequestration of rodent malaria parasite in lungs and adipose tissues [17]. This is not surprising given that CD36 is a multiligand scavenger receptor and mediates binding and uptake of a wide variety of particulate ligands such as oxidized low-density lipoproteins, β-amyloid plaque, bacteria, and apoptotic cells by macrophages [18], [19].

In the case of malaria, CD36 functions as a main receptor for the adherence of IRBCs and consequent sequestration of parasites in the microvascular endothelia [7]–[10]. CD36 also controls parasitemia through phagocytic clearance of IRBCs by macrophages and protects mice against malaria [20]–[23]. Furthermore, *Cd36* mutations in endemic population have been shown to contribute to either protection from severe malaria or susceptibility to illness [24]–[27], which presumably depends on host factors and infection dynamics. Studies have reported that CD36 mediates the binding of *P. falciparum* IRBCs to human monocyte-derived DCs, but the binding rendered DCs to be immunosuppressive, i.e., cells produce little or no TNF-α and IL-12 in response to IRBCs or subsequent stimulation with LPS [28], [29]. Additionally, ongoing studies by us and previous studies by others have shown that the uptake of *P. falciparum* IRBCs produces little or no pro-inflammatory cytokines by human and mouse macrophages [21], [30], [31], [unpublished results]. Thus, the cellular and molecular basis for the CD36-dependent development of immunity to malaria remains not understood.

Recent studies have shown that human blood DCs, mouse spleen DCs, and FL-DCs and GM-DCs obtained by the differentiation of mouse bone marrow cells by FLT3 ligand and GM-CSF, respectively, robustly produce pro-inflammatory cytokines in response to IRBCs [32]–[37], (reviewed in [38]). DCs from the spleens of malaria parasite-infected mice activate T cells to efficiently induce cytokine responses [39]. Considering that DCs represent a critical component of the immune system, and that these cells are not only important for the early cytokine responses but also essential for bridging and regulating the innate and adaptive immune responses to pathogenic infections [40], [41], we hypothesize that CD36 contributes to malaria immunity. Accordingly, we studied the role of CD36 in the uptake of *P. falciparum* IRBCs and the production of pro-inflammatory cytokine by human and mouse DCs. Additionally, we studied the ability of IRBC-activated DCs to stimulate NK and T cells to produce IFN-γ. These results, for the first time, unambiguously show that CD36 plays an important role in pro-inflammatory cytokine responses and other DC functions.

## Materials and Methods

### Reagents

CpG ODN-1826 was from Coley Pharmaceutical Canada (Kanata, ON, Canada) and Cell Sciences (Canton, MA), respectively. LPS was from Sigma-Aldrich (St. Louis, MO). Cell Trace™ CFSE cell-staining kit was from Molecular Probes, Inc. (Eugene, OR). ELISA kits for analysis of human and mouse TNF-α, and mouse IL-12p40 and IFN-γ were from R&D Systems (Minneapolis, MN). The ELISA kit to assay human IL-12 was from PeproTech (Rocky Hill, NJ). Anti-mouse NK cell isolation kit, anti-mouse CD90.2 antibody conjugated microbeads, human blood DC isolation kit II, and magnetic columns for cell separation, fluorescein isothiocyanate (FITC)-conjugated anti-human CD1c antibody (clone AD5-8E7), and allophycocyanin (APC)-conjugated anti-human CD304 (BDCA-4/neuropilin-1, clone AD5-17F6) were from Miltenyi Biotec Inc. (Auburn, CA). Mouse monoclonal antibody against mouse CD36 (JC63.1) was from Cayman Chemical (Ann Arbor, MI). Anti-mouse CD16/32 monoclonal antibody (clone 93), FITC-conjugated antibodies against mouse pan-NK cells (DX5), and mouse CD3ε (145-2C11), phycoerythrin (PE)-conjugated anti-mouse IgA (11-44-2), and anti-mouse NK1.1 (PK136), PE-Cy7-labeled rat anti-mouse IgG1 (M1-14D12), peridinin-chlorophyll-protein (PerCP)-Cy5.5-conjugated antibody against mouse CD11b (M1/70) and APC-conjugated antibody against human CD1c (L161), and the isotype controls mouse IgA and mouse IgG1 were from eBioscience (San Diego, CA). Mouse monoclonal antibody against human CD36 (FA6-152) was from Immunotech, Beckman Coulter Inc. (Brea, CA). APC-conjugated antibody against mouse CD11c (418N) was from BD Biosciences (San Jose, CA). The Chinese hamster ovary (CHO)-745 mutant cells that stably express human CD36, but deficient in the expression of C4S [10], were provided by Professor Artur Scherf, Pasteur Institute, Paris, France. Fms-like tyrosine kinase 3 (FLT-3) ligand expressing B16 cell line [42] was provided by Dr. Glenn Dranoff, Dana-Farber Cancer Institute, Boston, MA.

### Mice

All mice used in this study were in C57BL/6J background and were housed in a pathogen-free environment. The animal care was in accordance with the institutional guidelines of the Hershey Medical Center, Hershey, Pennsylvania.

### Ethics Statement

The Institutional Review Board (IRB) of the Pennsylvania State University College of Medicine, Hershey, Pennsylvania, has reviewed and approved the procedure for collection of blood from healthy, adult volunteers and informed consent form (protocol No. HY03-261). Blood was drawn after informed consent form signed by volunteers. The human plasma was purchased from the Blood Bank, Hershey Medical Center, Hershey, and the IRB of the Pennsylvania State University College of Medicine, Hershey, has approved its use for culturing *P. falciparum*.

The animal studies were performed in accordance with the recommendations in the Guide for the Care and Use of Laboratory Animals of the National Institutes of Health. The Institutional Animal Care and Use Committee (IACUC) of the Pennsylvania State University College of Medicine, Hershey, has reviewed and approved the protocols for the use of mice (No. 2001-146).

### Parasite Culturing

*P. falciparum* (3D7 strain) was cultured using O-positive human erythrocytes in RPMI 1640 medium containing 10% human O-positive plasma as described previously [43]. The cultures were free from mycoplasma contamination. The trophozoite stage IRBCs were enriched on 70% percoll cushions as described previously [32].

### Selection of CD36-adherent and CD36-nonadherent IRBCs

The CD36-adherent and CD36-nonadherent IRBCs were selected as described previously [43], [44]. Briefly, the early to mid trophozoite stage IRBCs from parasite cultures were overlaid onto the monolayers of human CD36-expressing CHO-745 cells and cultured as described previously in 25 cm^2^ culture flasks for 45 min [10]. The non-adherent IRBCs were removed by washing with RPMI 1640 medium. RBCs were added to the monolayers of CHO-745 cells containing bound IRBCs and further cultured. Soon after the merozoites invaded RBCs, the early ring stage IRBCs (before they become CD36-adherent) were harvested, cultured and re-selected for CD36 adherence. The selection process was repeated until all the CD36-nonadherent IRBCs were removed. The CD36-nonadherent IRBCs were obtained by the depletion of CD36-adherent IRBCs on monolayers of the CHO-745 cells. The CD36-adherent and CD36-nonadherent IRBCs thus obtained were cultured for 3–4 life cycles and percoll-enriched IRBCs at the late trophozoite stage IRBCs were used for cell stimulation.

### CFSE Staining of IRBCs

The percoll-enriched IRBCs and control RBCs (1×10^7^ cells/ml), suspended in PBS, pH 7.2, were stained with 2 µM of CFSE at 37°C in the dark. After 10 min, 2 volumes of FBS was added and incubated at room temperature for 10 min. The cells were washed three times with PBS.

### Isolation and Analysis of Human Blood DCs

Peripheral blood mononuclear cells (PBMCs) from the Buffy coat of healthy human blood were isolated by centrifugation on ISOLYMPH (CTL Scientific Supply, Deer Park, NY) cushions [32]. DCs were isolated from PBMCs by magnetic separation using human blood DC isolation kit II according to manufacturer’s instruction; the purity of cells was about 60%. The purified DCs were surface stained with anti-CD1c and anti-CD304 antibodies and analyzed by using a Becton-Dickinson FACSCalibur flow cytometer. Unless otherwise stated, the results were analyzed using CellQuest software (BD Biosciences, San Jose, CA).

### Preparation of FLT3 Ligand Differentiated DCs

Bone marrow cells from WT and *Cd36* ^−/−^ mice were cultured for 7 to 8 days in complete DMEM supplemented with 15% of conditioned medium from FLT3 ligand expressing B16 cells [42], [45]. These DCs were designated as FL-DCs.

### Isolation of Spleen Cells

Single cell suspensions of mouse spleens were prepared as described previously [32]. From this preparation, NK cells were isolated by magnetic separation using NK cell isolation kits; the purity was ∼60%. T cells were isolated using anti-mouse CD90.2 antibody-conjugated magnetic beads; the purity was ∼90%.

### Analysis of CD36 Expression on the Surface of DCs

Human blood DCs suspended in PBS containing 1% BSA were stained with dye-conjugated anti-human CD1c and anti-CD304 monoclonal antibodies at 4°C for 10–15 min. The cells were also stained with anti-human CD36 monoclonal IgG1 followed by dye-conjugated anti-mouse IgG1 as a secondary antibody. A mouse IgG1 was used as an isotype control. The cells were analyzed by flow cytometry and the results were analyzed using FlowJo software (Tree Star, Ashland, OR). Similarly, mouse DCs were stained with dye-conjugated anti-mouse CD11c and anti-mouse CD11b antibodies and with anti-mouse CD36 IgA followed by dye-conjugated rat anti-mouse IgA secondary antibody. A non-specific mouse IgA was used as isotype control. The cells were analyzed by flow cytometry.

### Analysis of IRBC Uptake by DCs

Human blood DCs were treated with either anti-human CD36 blocking antibody or an isotype control antibody (in each case 10 µg/ml) at 4°C for 10 min. The cells were seeded into 24-well plates (5×10^5^ cells/well in 0.3 ml/well complete DMEM) and incubated with percoll-enriched, CFSE stained IRBCs. After 2 h, DCs were stained with either anti-CD1c or anti-CD304 antibodies, and analyzed by flow cytometry. DCs incubated with uninfected RBCs were analyzed as controls. The internalization of CFSE-stained IRBCs by mouse FL-DCs was similarly analyzed.

### Cell Stimulation and Cytokine Analysis

Human DCs were either untreated or treated with 10 µg/ml of anti-human CD36 antibody at 4°C for 10 min. The washed cells were seeded into 96-well plates (1×10^5^ cells/well in 200 µl complete DMEM) and then stimulated with IRBCs or LPS control for 24 h. Mouse FL-DCs in 96-well plates (1×10^5^ cells/well in 200 µl complete DMEM) were stimulated with IRBCs or CpG control for 24 h. The culture supernatants were collected and cytokines measured by ELISA [46].

For NK cell activation, FL-DCs (1×10^5^ cells/well) and NK cells (5×10^4^ cells/well) were co-cultured in U-bottom 96-well plates and stimulated with CD36-adherent or CD36-nonadherent IRBCs (1×10^5^ cells/well). After 36 h, the culture supernatants were harvested and analyzed for IFN-γ by ELISA. FL-DCs or NK cells alone stimulated similarly were used as controls.

For T cell activation, FL-DCs in 96-well U-bottom plates (1×10^5^ cells/well) were incubated with the indicated doses of CD36-adherent IRBCs for 6 h and then co-cultured with spleen T cells (5×10^4^/well) from OT-II transgenic mice in the presence or absence of 2 µg/ml OVA^323–339^ peptide in 200 µl of complete medium. After 72 h, culture supernatants were harvested and assayed for IFN-γ by ELISA.

### Statistical Analysis

One-way analysis of variance followed by Newman-Keuls test was used to determine statistical significance between cytokine responses from different pairs of samples. The analysis was done using GraphPad prism version 3.0. *P* values <0.05 were considered statistically significant.

## Results

### CD36 Contributes to the Uptake of *P. falciparum* IRBCs and Cytokine Production by Human DCs

**Figure 1:**
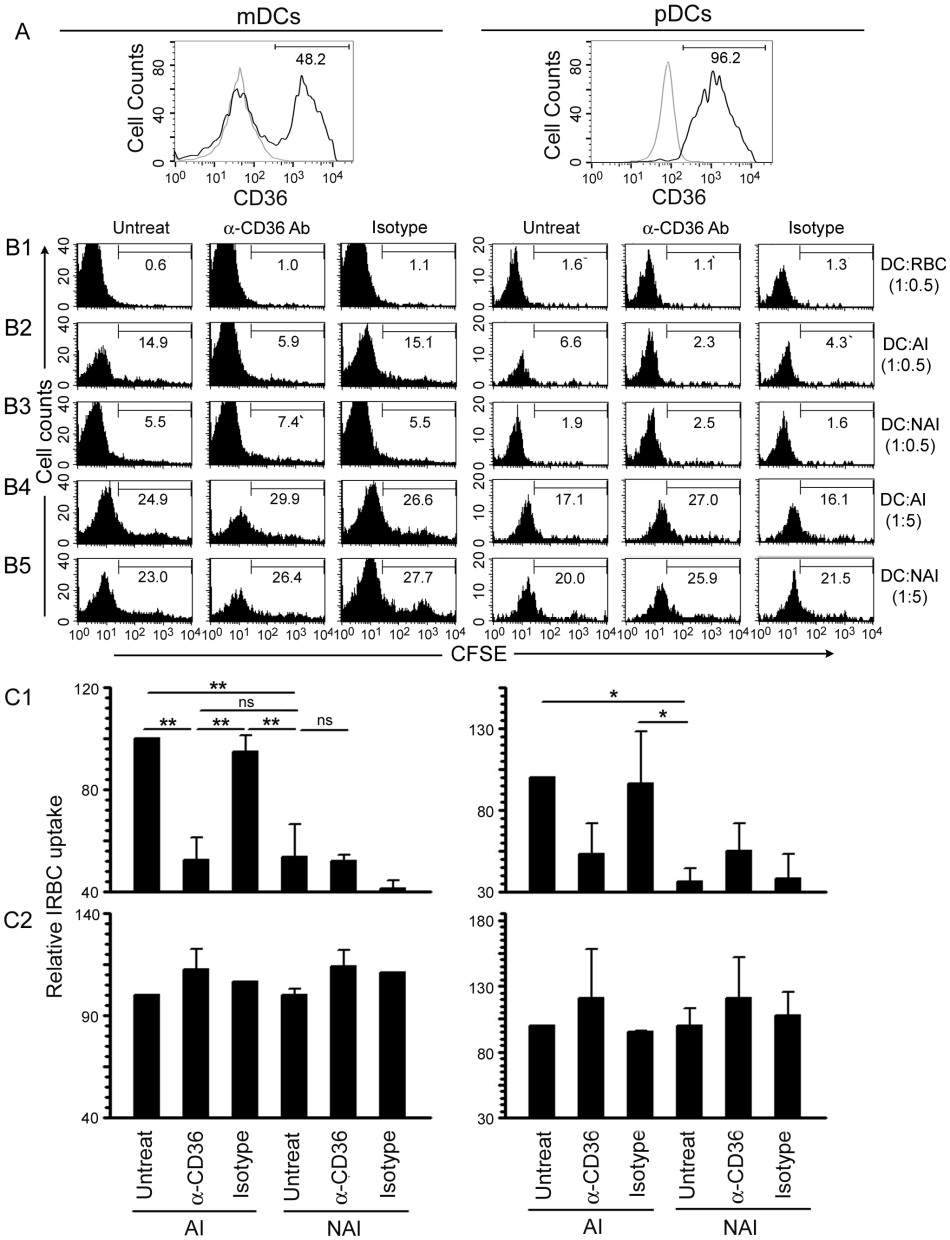
CD36 is expressed on human mDC and pDC surface and contributes to their IRBC uptake. **A**. The expression of CD36 on the surface of human blood DCs was assessed by flow cytometry using anti-human CD36 monoclonal antibody (black line) and monoclonal IgG1 isotype control (grey line). The values given in the histograms indicate the percent CD36-positive cells in mDC and pDC populations. **B1–B5**. Human blood DCs were treated with either anti-human CD36 monoclonal antibody or an isotype control and then incubated with CFSE-stained *P. falciparum* CD36-adherent IRBCs (AI) or CD36-nonadherent IRBCs (NAI) as outlined in Materials and Methods. The uptake of IRBCs was assessed by flow cytometry. DCs incubated with CFSE-treated RBCs at DC to RBC ratio of 1∶0.5 were analyzed as a control. The cells were stained with anti-human CD1c and CD304 antibodies. Shown are the results of IRBC uptake by the gated mDCs (CD1c^+^) and pDCs (CD304^+^) populations from one of three independent experiments. The values shown in the histograms indicate the percent CFSE-positive cells. **C1 and C2**. Plots of the relative IRBC uptake±SEM of three independent experiments performed using DC to IRBC ratios of 1∶0.5 (C1) and 1∶5 (C2). *, *p*<0.05; **, *p*<0.01, ns, not significant.

Malaria parasites induce strong pro-inflammatory cytokine responses in both human and mouse DCs predominantly through TLR9-mediated recognition [32], [39], [47]. Unlike in mice, where both myeloid DCs (mDCs) and plasmacytoid DCs (pDCs) express TLR9 [48], in humans, pDCs but not mDCs express TLR9 [49]–[51]. Consequently, human pDCs but not mDCs efficiently produce pro-inflammatory cytokines in response to malaria parasites, although interaction between both cell subpopulations is required for the robust production of cytokines such as IL-12 [32]. Therefore, we first assessed the surface expression of CD36 in human DCs using anti-human CD36 monoclonal antibody. The data showed that both CD1c(BDCA-1)^+^ mDCs and CD304(BDCA-4)^+^ pDCs (present in ∼1.6∶1 proportion), which together accounts for >95% of the total human blood DCs [52], express substantial levels of CD36 on their surface (Figure 1A).

Next, to determine whether CD36 mediates the internalization of IRBCs by human mDCs and pDCs, we analyzed the uptake of CFSE-labeled *P. falciparum* IRBCs with and without prior blocking of CD36 with an anti-human CD36 antibody. The difference in the levels of internalized CD36-adherent and CD36-nonadherent IRBCs was considered as the measure of the CD36-dependent IRBC uptake. In the absence of antibody and at relatively lower IRBC doses, mDCs and pDCs internalized significantly higher levels of CD36-adherent IRBCs than CD36-nonadherent IRBCs (Figure 1C1, and compare column 1 in panels B2 and B3 of mDCs and pDCs in Figure 1). Upon prior blocking of CD36 with anti-CD36 antibody, the uptake of CD36-adherent IRBCs by mDCs and pDCs was significantly lower (Figure 1C1, and compare columns 1 and 2 of mDCs and pDCs in Figure 1B2). However, there was no significant change in the uptake of CD36 adherent IRBCs by DCs treated with the isotype control antibody (Figure 1C1, and compare columns 1 and 3 in Figure 1B2). Furthermore, there was no significant difference in the uptake of CD36-nonaherent IRBCs by mDCs and pDCs upon prior blocking of CD36 with either anti-CD36 antibody or isotype control antibody (Figure 1C1, and compare columns 1 and 2, and columns 1 and 3 in Figure 1B3). Moreover, there was no significant difference in the uptake of non-adherent IRBCs by mDCs or pDCs compared to the internalization of CD36-adherent IRBCs by anti-CD36 antibody-treated DCs (Figure 1C1, and compare column 1 in B3 with column 2 in B2 of mDCs and pDCs). Together, these results indicate that CD36-mediated binding contributes substantially to the uptake of *P. falciparum* IRBCs by human DCs.

At higher IRBC doses, there was no significant difference in the uptake of CD36-adherent and CD36-nonadherent IRBCs by either mDCs or pDCs (Figure 1C2, and compare column 1 in panels B4 with that in B5). This is likely due to the increased rate of IRBC uptake mediated by other scavenger phagocytic receptors, thereby masking the CD36-mediated uptake. Although some increase in the uptake of CD36-adherent or CD36-nonadherent IRBCs was observed when CD36 on DCs was blocked with anti-CD36 antibody (compare columns 1 and 2 in Figure 1B4 and 1B5, and also see Fig. 1B3), this was not statistically significant (Fig. 1C). Thus, at high IRBC doses, which are unlikely to be biologically relevant as parasitemia is low at early stages of infection, it is not possible to distinguish between the CD36-dependent and CD36-independent uptake mechanisms.

**Figure 2:**
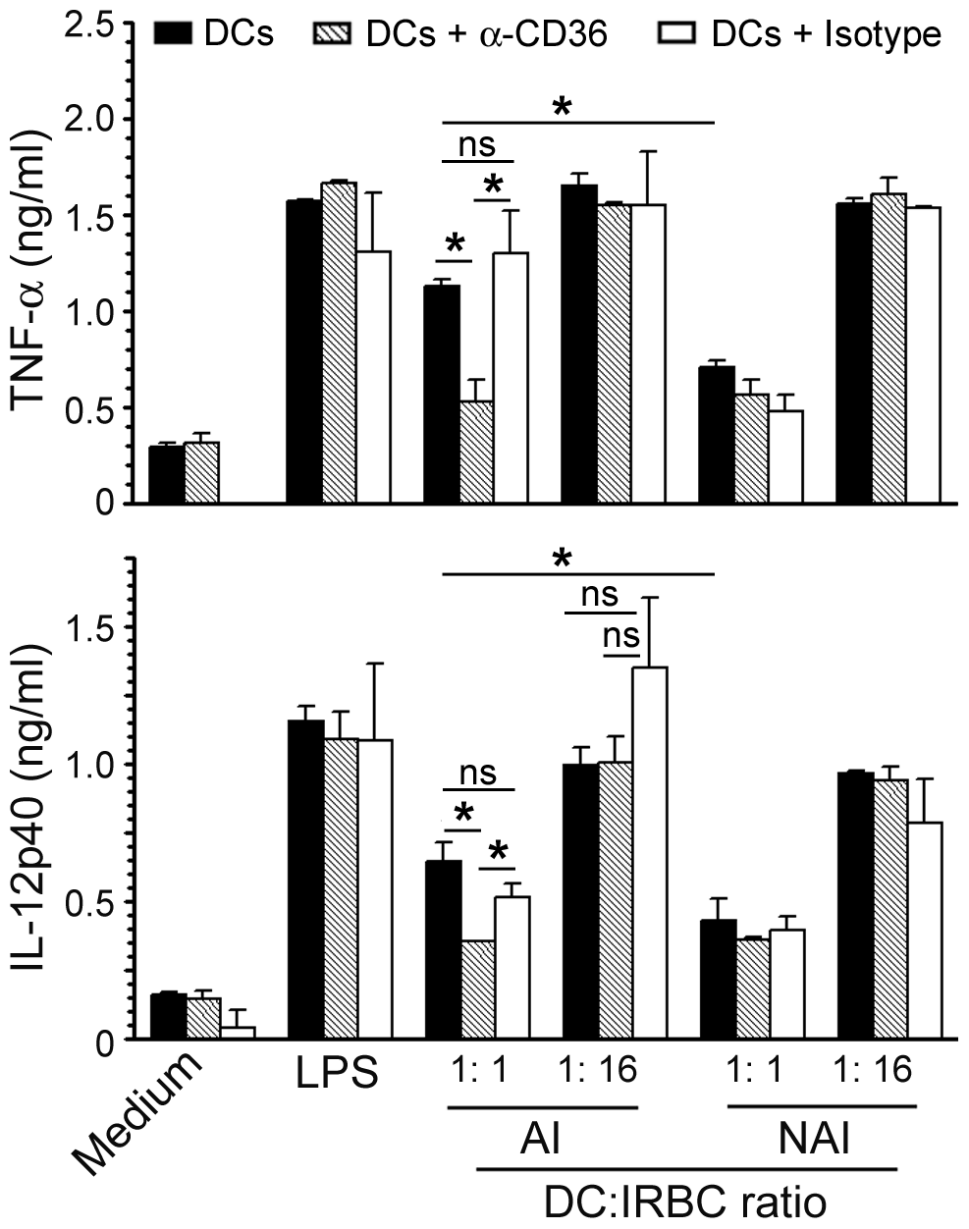
CD36 contributes to pro-inflammatory cytokine production by DCs in response to malaria parasites. Human DCs were either untreated or treated with anti-human CD36 antibody or isotype control antibody and then stimulated with the indicated doses of *P. falciparum* CD36-adherent IRBCs (AI) or CD36-nonadherent IRBCs (NAI) for 24 h. DCs stimulated with LPS (100 ng/ml) was used as a control. TNF-α and IL-12 secreted into the culture media were measured by ELISA. Shown are the data from a representative of at least three independent experiments. Mean values ± SD are plotted. *, *p*<0.05; **, *p*<0.01; ns, not significant.

Given that CD36 is physically associated with the Src and Syk family kinases [53], and that CD36-mediated phagocytosis initiates Src/Syk signaling [54]–[56], which may synergize with TLR-induced signaling by pathogens, we hypothesized that CD36-dependent uptake and signaling together contribute to cytokine production induced by IRBCs. Thus, we analyzed pro-inflammatory cytokine responses to CD36-adherent and CD36-nonadherent IRBCs by human blood DCs. Considering our previous observation that the cooperation between human pDCs and mDCs is required for the robust cytokine responses [32], we analyzed cytokine production by total DCs. At low IRBC doses, in agreement with the observed difference in CD36-dependent uptake of IRBCs (see Figure 1), the production of TNF-α and IL-12 by DCs stimulated with CD36-adherent IRBCs was significantly higher than that by DCs stimulated with CD36-nonadherent IRBCs (Figure 2). This CD36-dependence was not observed when cells were stimulated with LPS. Prior blocking of CD36 with anti-human CD36 antibody resulted in a significant (∼50%) decrease in the production of TNF-α and IL-12 by DCs in response to CD36-adherent IRBCs but not to CD36-nonadherent IRBCs, whereas treatment with istotype control antibody had no effect (Figure 2). Together these data demonstrate that CD36 plays an important role in IRBC-induced pro-inflammatory cytokine production. Of note is that at higher IRBC doses, there was no significant difference in the production of TNF-α or IL-12 in response to CD36-adherent and CD36-nonadherent IRBCs (Figure 2). This is consistent with the observed high levels of IRBC uptake through both CD36-dependent and CD36-independent mechanisms, thereby masking the CD36-mediated uptake (see Figure 1).

### Mouse CD36 Contributes Phagocytic uptake of *P. falciparum* IRBCs by DCs

To further demonstrate the role of CD36 in the IRBC uptake and cytokine responses by DCs, we studied FL-DCs from WT and *Cd36^−/−^* mice. Although *P. falciparum* does not infect mice, mouse and human CD36 closely resemble one another (90% identity in their amino acid sequences), and that mouse CD36 mediates the sequestration of rodent malaria parasites *in vivo* [17]. Mouse CD36 is also known to contribute to the uptake of *P. falciparum* IRBCs by macrophages [20], [21]. Therefore, mouse cells are relevant to study the role of CD36 in immune responses to IRBCs.

**Figure 3:**
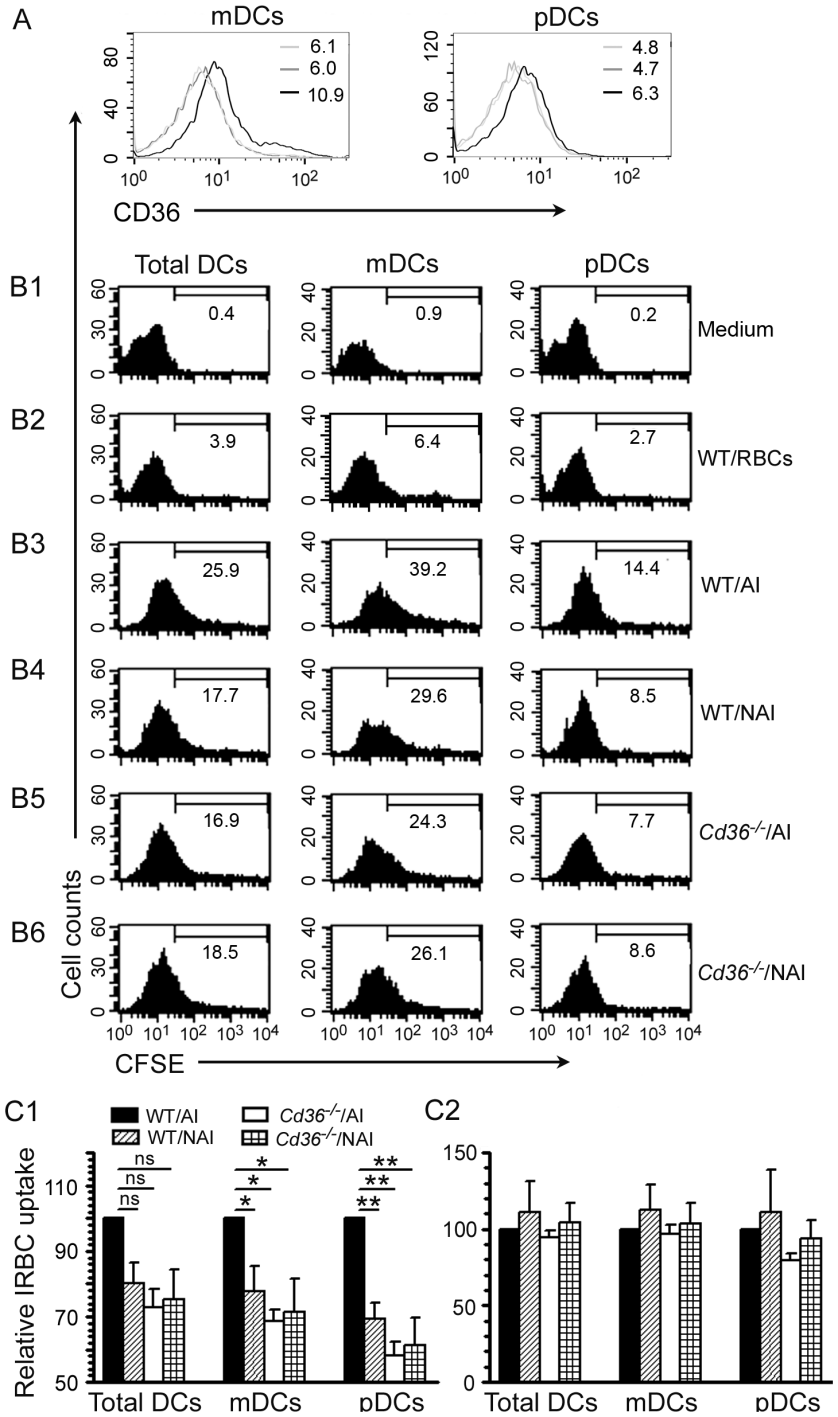
Mouse DCs express CD36 on the surface and internalize IRBCs in a CD36-dependent manner. **A**. The expression of CD36 on the surface of mDC and pDC populations of mouse FL-DCs as assessed by flow cytometry after staining with anti-mouse CD36 monoclonal antibody. In both mDC and pDC panels, black line refers to WT DCs treated with anti-CD36-antibody, grey line indicates CD36 deficient DCs treated with anti-CD36 antibody, and light gray line refers to WT DCs treated with a monoclonal IgA isotype control. The values shown in the histograms are geometric mean fluorescent density. **B1–B6**. FL-DCs from WT and *Cd36* ^−/−^ mice were incubated with CFSE-stained *P. falciparum* CD36-adherent IRBCs (AI) or CD36-nonadherent IRBCs (NAI) at DC to IRBC ratio of 1∶0.5. DCs incubated with mock CFSE-stained RBCs at DC to RBC ratio of 1∶0.5 were analyzed as control. The uptake of IRBCs was assessed by flow cytometry. The values given in the histograms indicate percent CFSE-positive cells. The data shown are from a representative of three independent experiments. **C1 and C2**. Plots of the relative IRBC uptake±SEM of three independent experiments performed using DC to IRBC ratios of 1∶0.5 (**C1**) and 1∶5 (**C2**). *, *p*<0.05; **, *p*<0.01; ns, not significant.

First, we tested the expression of CD36 on the surface of mouse FL-DCs using antibody against mouse CD36. FL-DCs consist of equal proportions of mDCs and pDCs [32]. Both mDCs and pDCs expressed CD36 on their surfaces (Figure 3A), although to a lesser extent compared to human blood DCs. Next, we measured the CD36-dependent uptake of IRBCs by FL-DCs using CFSE-labeled IRBCs. At low IRBC doses, the uptake of CD36-nonadherent IRBCs by WT FL-DCs was significantly lower than that of CD36-adherent IRBCs (Figure 3C1, and compare column 1 in B4 with that in B3). Similarly, the uptake of CD36-adherent IRBCs by *Cd36^−/−^* FL-DCs was lower than that by WT DCs (Figure 3C1, and compare column 1 in B5 with that in B3). In the case of CD36-nonadherent IRBCs, there was no significant difference in the level of IRBC internalization by DCs from WT and *Cd36^−/−^* mice (Figure 3C1, and compare column 1 in B6 with that in B4). Furthermore, the uptake of both CD36-adherent and CD36-nonadherent IRBCs by *Cd36^−/−^* DCs was nearly comparable (Figure 3C1, and compare column 1 in B5 with that in B6). Together these results indicate that mouse CD36 also contribute to the uptake of IRBCs by DCs.

We also analyzed the IRBC uptake by FL-DCs at higher IRBC doses (DC to IRBC ratios of 1∶5). Both WT and *Cd36* ^−/−^ DCs internalized IRBCs to comparable extents irrespective of whether IRBCs were CD36-adherent or CD36-nonadherent (Figure. 3C2). This is likely because, as mentioned in the case of human DCs, when IRBCs are in large excess, uptake *via* CD36-independent mechanisms masks the CD36-dependent uptake.

### CD36 Contributes to IRBC-induced Inflammatory Cytokine Responses by Mouse DCs

**Figure 4:**
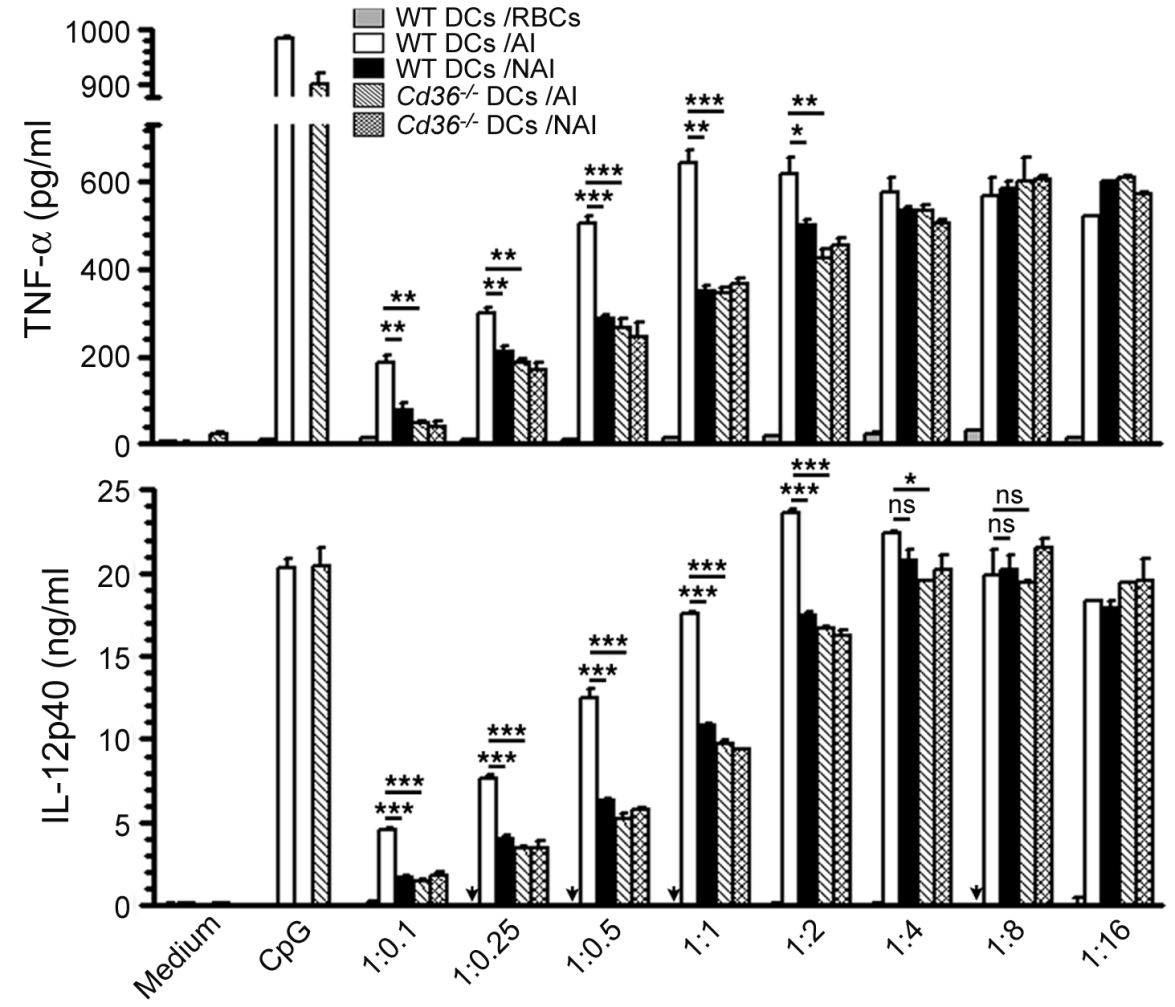
CD36 contributes to IRBC-induced pro-inflammatory cytokine production by DCs. FL-DCs from WT and *Cd36* ^−/−^ mice were stimulated with the indicated doses of CD36-adherent IRBCs (AI) and CD36-nonadherent IRBCs (NAI) for 24 h. WT DCs stimulated with RBCs, and WT DCs and *Cd36^−/−^* DCs stimulated with CpG (2 µg/ml) were analyzed as controls. TNF-α and IL-12 secreted into the culture media were measured by ELISA. The experiments were performed three times. Shown are results from one of three independent experiments. Mean values ± SD are plotted. *, *p*<0.05; **, *p*<0.01; ***, *p*<0.001; ns, not significant. IL-12 was not detectable (indicated by arrows) in WT FL-DCs treated with RBCs.

To determine whether the CD36-dependent IRBC uptake by DCs is reflected in their cytokine production, we measured TNF-α and IL-12 responses to CD36-adherent and CD36-nonadherent IRBCs by FL-DCs from WT and *Cd36* ^−/−^ mice. At lower IRBC doses, the production of TNF-α and IL-12 by WT FL-DCs stimulated with CD36-adherent IRBCs was significantly higher than that by WT FL-DCs stimulated with CD36-nonadherent IRBCs (Figure 4). Also, at similar doses, FL-DCs from *Cd36* ^−/−^ mice produced significantly lower levels of TNF-α and IL-12 in response to CD36-adherent IRBCs compared to WT FL-DCs. As expected based on the observed similar levels of IRBC uptake at higher doses (see Figure 3C2), irrespective of whether IRBCs were CD36-adherent or not, TNF-α and IL-12 responses by FL-DCs from both WT and *Cd36* ^−/−^ mice were comparable (Figure 4). Together, these data demonstrate that CD36 contributes to the production of pro-inflammatory cytokine by DCs.

### Cytokine Production by NK and T Cells in Response to IRBC-activated DCs is CD36-Dependent

**Figure 5:**
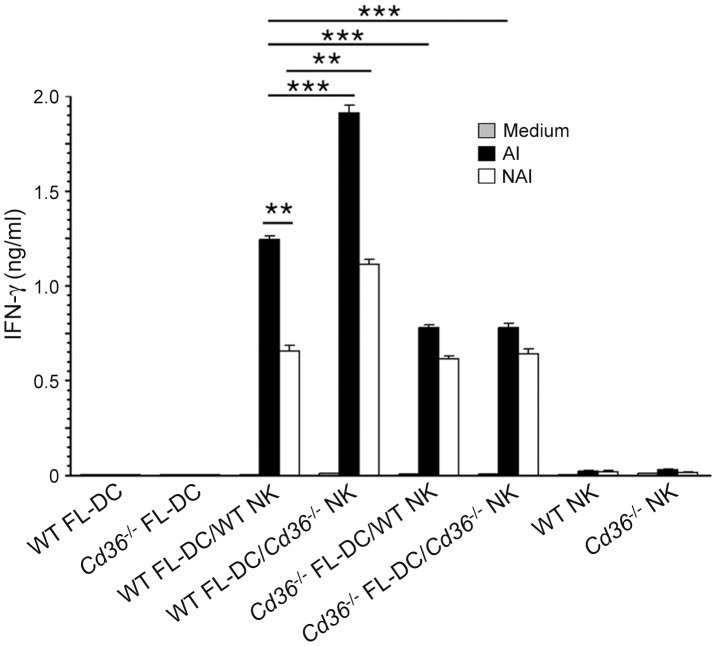
CD36 contributes to the IFN-γ production by NK cells stimulated with IRBC-treated DCs. NK cells from WT and *Cd36* ^−/−^ mice co-cultured with WT and *Cd36* ^−/−^ FL-DCs and then stimulated with CD36-adherent IRBCs (AI) or CD36-nonadherent IRBCs (NAI) for 36 h. DCs and NK cells alone stimulated with IRBCs were used as controls. IFN-γ secreted into the culture media was analyzed by ELISA. Experiments were performed three times and shown are the results of one representative experiment. Mean values ± SD are plotted. **, *p*<0.01; ***, *p*<0.001.

Since CD36 contributes to the IRBC-induced pro-inflammatory cytokine production by DCs, it was of interest to determine whether the CD36-dependent programming of IRBC-induced function of DCs reflected in cytokine responses by NK and T cells. Incubation of the co-cultures of FL-DCs and NK cells with CD36-adherent IRBCs resulted in significantly lower production of IFN-γ by either WT or *Cd36* ^−/−^ NK cells activated by *Cd36* ^−/−^ DCs compared to that produced by WT NK cells stimulated by WT DCs (Figure 5, black bars). In contrast, in the case of DCs activated by CD36-nonadherent IRBCs, both WT and *Cd36^−/−^* NK cells stimulated with *Cd36^−/−^* DCs produced lower but similar levels of IFN-γ compared to that produced by WT NK cells stimulated with WT DCs (Figure 5, open bars). Interestingly, *Cd36* ^−/−^ NK cells, co-cultured with WT DCs and activated with either CD36-adherent or CD36-nonadherent IRBCs, produced significantly higher levels of IFN-γ than WT NK cells stimulated with similarly activated WT DCs. Although the reason for this unexpected observation remains to be studied, the above results demonstrated that CD36 significantly contributes to the malaria parasite-induced IFN-γ production by NK cells.

**Figure 6:**
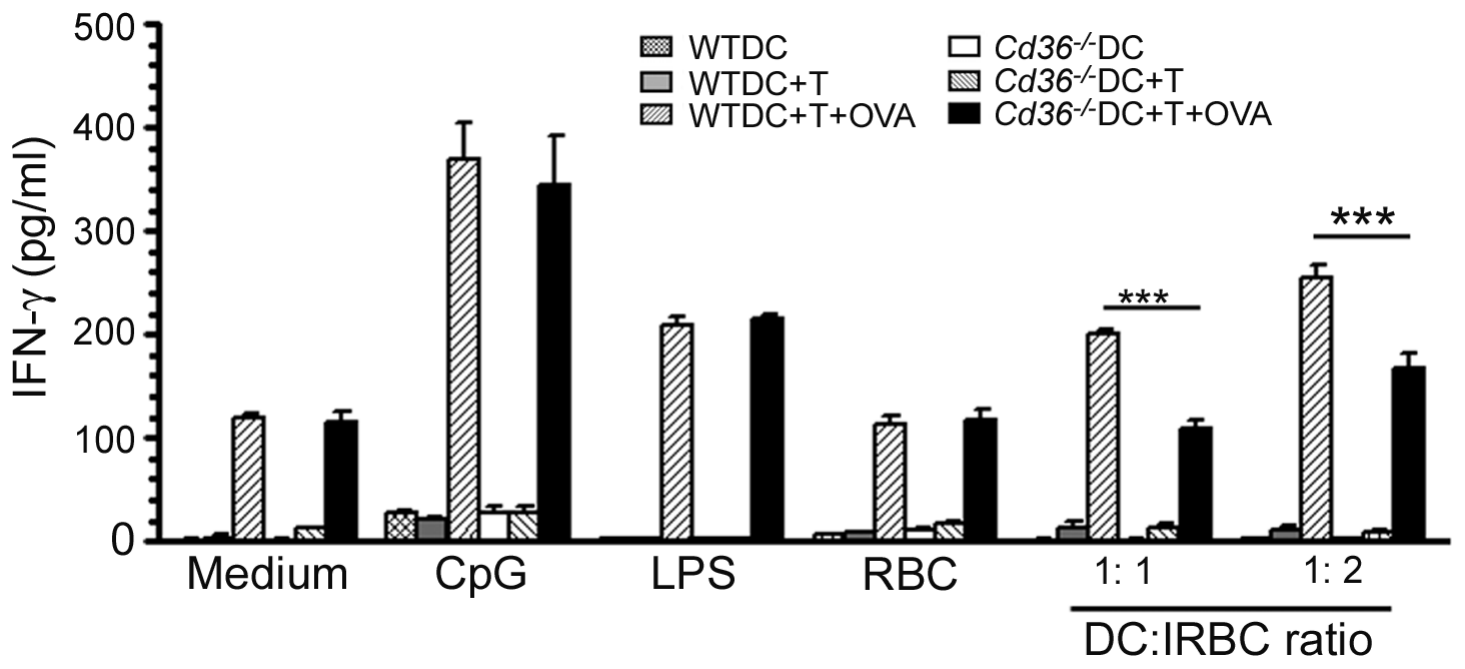
CD36 contributes to IFN-γ production by T cells stimulated with IRBC-treated DCs. FL-DCs from WT and *Cd36* ^−/−^ mice were stimulated with CD36-adherent IRBCs for 6 h and then co-cultured with T cells from OT-II transgenic mice in the presence of OVA^323–339^ peptide for 72 h. Untreated DCs and DCs stimulated with RBCs, LPS (100 ng/ml) or CpG (2 µg/ml) were used as controls. DCs cultured alone or co-cultured with OT-II T cells and stimulated with IRBCs in absence of OVA^323–339^ peptide were also used as controls. IFN-γ secreted into the culture media was analyzed by ELISA. Data are a representative of three independent experiments. Error bars represents mean values ± SD. ***, *p*<0.001.

To determine whether CD36 influences the ability of DCs to induce cytokine responses by T cells, we co-cultured OT-II T cells with FL-DCs treated with CD36-adherent IRBCs in the presence of OVA peptide. OT-II T cells stimulated with *Cd36^−/−^* DCs produced significantly lower levels of IFN-γ than OT-II T cells stimulated with WT DCs at both doses of IRBCs tested (Figure 6), indicating that CD36 deficiency in DCs leads to decreased cytokine production by T cells. Thus, these results demonstrate that CD36 contributes to the ability of DCs to induce IFN-γ responses in T cells.

## Discussion

CD36 has been shown to contribute to inflammatory responses to various pathogens and to endogenous pathogenic components such as β-amyloid plaque and low-density lipoprotein complex through the receptor-mediated activation of mitogen-activated protein kinase (MAPK) and NF-κB signaling pathways, leading to high levels of inflammation and pathogenesis of diseases such as Alzheimer’s disease, atherosclerosis, diabetes, and infectious diseases [19], [20], [57], [58]. However, the role of CD36 in the development of immunity to malaria remains poorly understood. Here, we show that the CD36-mediated uptake of IRBCs plays an important role in the pro-inflammatory cytokine responses to malaria by DCs and in the DC-dependent activation of NK and T cells and cytokine production. This is evident from our observation that the uptake of IRBCs and consequent production of pro-inflammatory cytokines by human and mouse DCs substantially dependent on the CD36-adherent property of IRBCs. Importantly, our observations that NK and T cells stimulated by CD36-adherent IRBC-activated WT DCs produce substantially higher levels of IFN-γ than those stimulated with similarly activated *Cd36* ^−/−^ DCs support the notion that CD36 plays an important role in malaria immunity. Based on the pattern of IRBC uptake, it is possible that other non-specific scavenger receptors also modulate immune response to malaria to certain extent. This is not surprising given that malaria parasites are known to interact with host through redundant mechanisms.

Our findings that, at low parasite doses, which likely resemble the situation during early stages of malaria infection having low parasitemia, CD36 contributes substantially to both IRBC uptake and cytokine production suggest that CD36 significantly influences malaria immunity at the early stage of infection. Thus, it is tempting to predict that the effect induced by CD36 early on during infection leads to programming of DCs to modulate immunity to malaria. We recently showed that an efficient production of pro-inflammatory cytokines during the early stages of infection is critical for the effective development of protective immunity to malaria and that DCs play a crucial role in these processes [39]. These observations together with our findings here that CD36 contributes to the parasite uptake and pro-inflammatory cytokine production indicate that CD36 modulates the ability of DCs to regulate immune responses to malaria. Consistent with this idea, in a mouse malaria infection model, the deficiency in CD36 was found to be associated with significantly lower inflammatory cytokine responses, lower parasite clearance, elevated parasitemia, and increased malaria severity and fatality [22].

CD36 plays dual roles in malaria infection, especially in the case of *P. falciparum* infection. On one hand, CD36-depedent sequestration of IRBCs in the microvascular capillaries of organs contributes to fatal conditions by exacerbating inflammation through enhanced production of pro-inflammatory cytokines locally and disruption of endothelial barrier, causing organ dysfunction [4]–[8]. On the other hand, the contribution of CD36 toward the production of pro-inflammatory cytokines is essential for the efficient development of protective immunity to malaria [20]–[22]. The harmful and protective dual roles of CD36 are also evident from the results of human population studies [24]–[27]. People in malaria endemic areas have been shown to have a relatively high frequency of *Cd36* mutations and that these mutations contribute differently to the outcome of malaria infection. Studies in Thailand population demonstrated that *Cd36* polymorphism was associated with protection from cerebral malaria [24], [25]. In African population, while one study found that a nonsense mutation in *Cd36* provided protection from severe malaria such as severe anemia, respiratory distress and hypoglycemia [27], another study found that mutations in *Cd36* is associated with susceptibility to cerebral and other severe malaria conditions [26]. Even though the results of the population studies appear contradictory at first glance, it is likely that the observed CD36-dependent protective or detrimental effect is dependent on the host genetic background, immune status/previously acquired immunity, and parasite adherence specificity and growth rate. It is possible that these factors exert varied effects in different endemic settings, thereby influencing the role of CD36 in the outcomes of malaria infection. Additionally, people in malaria endemic areas are also exposed to bacterial, viral and other pathogenic infections, including tuberculosis, HIV, *Leishmania*, and parasites of the genus *Trypanosoma* and *Schistosoma* [59]–[62]. These infections may also skew the functions of DCs, leading to varying responses. In any event, although the CD36 functional deficiency in different populations may cause different effects in response to malaria infection, the results of all these studies are consistent with our conclusion here that CD36 significantly modulates immune responses to malaria.

Our finding that human pDCs are substantially less phagocytic than mDCs, even though pDCs express substantial levels of CD36 on their surface, appears to have an important implication from the point of view of malaria immunity. Thus, a question arises as to what is the biological significance of pDCs expressing a high level of CD36 although these cells are not meant for phagocytic clearance of parasites? We speculate that high-level expression of CD36 is an adaptive mechanism developed by pDCs in response to the co-existence of malaria parasites and other TLR9-activating pathogens during the course of human evolution for the efficient recognition of parasites and thereby contributing to the innate cytokine responses and subsequent adaptive immunity to malaria. This prediction is supported by our recent findings that, in *P. falciparum* and *P. yoelii*, nucleosomes/DNA are the dominant immunostimulatory component that activate DCs to induce pro-inflammatory cytokines and confer DCs the ability to activate NK cells through TLR9-mediated activation [32], [39], [47]. As mentioned above, human pDCs but not mDCs express TLR9 and produce IFN-α and TNF-α in response to malaria parasites and that the robust production of IL-12 requires cooperation of both pDCs and mDCs [32], [49]–[51]. Therefore, in the case of humans, we speculate that mDCs are mainly involved in the uptake of parasites and in the processing and presenting of antigens, whereas pDCs regulate innate and adaptive immunity to malaria through the production of pro-inflammatory cytokines. Since the cytokine milieu produced in response to infections by pathogenic organisms is known to profoundly influence the quality and effectiveness of adaptive immune responses, it is not surprising that, in response to the coexistence of malaria parasites with humans during the course of evolution and because of the selective pressure by TLR9-activating pathogens, pDCs evolved to express TLR9 and high levels of CD36 for the effective development of immunity to pathogens, including malaria. It would be interesting to determine the molecular mechanisms and signaling events involved in the CD36-mediated modulation of malarial immunity and accordingly it is our future goal.

In conclusion, in this study we demonstrate that CD36 plays a significant role in the uptake of malaria parasite IRBCs. CD36 also contributes significantly to inflammatory cytokine responses by DCs and to DC-induced cytokine responses by NK and T cells. The IRBC-dose dependent pro-inflammatory cytokine responses suggests that CD36 regulate malaria immunity at the early stages of infection when IRBC load is low, and that the early immune responses appear to substantially influence the subsequent development of malaria immunity. Our results also points to an interesting notion that the expression of high levels of CD36 and selective expression of TLR9 by human pDCs is an evolutionary adaptation for the effective immune responses to malaria during the long course of their co-existence.
